# The *H19* locus: regulatory function of an imprinted non coding RNA in embryonic development

**DOI:** 10.1186/1756-8935-6-S1-P54

**Published:** 2013-03-18

**Authors:** Paul Monnier, Clémence Martinet, Luisa Dandolo

**Affiliations:** 1 Department of Genetics and Development, Institut Cochin, Paris, France

## Background

Although the *H19* gene was discovered in 1984, its precise function is still relatively poorly understood. It is located on mouse chromosome 7, close to the *Igf2* (insulin-like growth factor 2) gene, in an imprinted locus. The maternal allele expresses a 2.3kb non coding RNA, as well as a micro RNA, the miR-675. We showed that *H19* acts as a transregulator of an imprinted gene network (IGN) involved in growth control of the embryo [1]. A recent study described that the miR-675 is exclusively expressed in the placenta during development, where it controls growth probably by downregulating the expression of *Igf1r*[2]. However, this micro RNA is unable to control the IGN in the embryo, control that appears therefore to be exerted by the full-length form of *H19*. The main goal of our work was to understand molecular mechanisms that drive this control of the IGN by *H19*.

## Materials and methods

Loss-of-function (*H19^Δ3^*) and gain-of-function (*H19^Tg^*) mouse mutants were used. *H19^Δ3/^*^+^*^;Tg^* females were crossed with *Mus musculus molossinus* (JF1) *wt* males. Polymorphisms between molossinus and domesticus mice allow to distinguish the maternal from the paternal allele. *H19^Δ3/^*^+^*^mol^*, *H19*^+^*^dom/^*^+^*^mol^* and *H19^-/^*^+^*^mol;Tg^* E14.5 embryos were collected from this cross in order to dissect limb muscles, or produce primary mouse embryonic fibroblasts (MEF) for ChIP and RIP (RNA immunoprecipitation) experiments.

## Results

We first investigated the imprinting status of the IGN and observed that *H19* controls by a *trans* mechanism the imprint of the *Igf2* gene, but not of other genes of the IGN. We performed RIP experiments in MEF and identified the MBD1 protein as a partner of the *H19* RNA. We also observed that some genes of the IGN, including the *Igf2* gene, are overexpressed both in *Mbd1^-/-^* and *H19^Δ3/^*^+^*^mol^* MEF. This suggests that *H19* may act on this network because of its interaction with the MBD1 protein. By ChIP experiments, we showed that MBD1 indeed binds to the *Igf2* gene and to other common targets of both *H19* and MBD1. Interestingly, this binding is lost in *H19^Δ3/^*^+^*^mol^* MEF. This indicates that *H19* is necessary for the recruitment of MBD1 to its targets.

## Conclusion

These results strongly suggest that the control of the expression of some genes of the IGN, including the *Igf2* gene, could be exerted through an interaction of the *H19* full-length RNA with the MBD1 protein.
